# Supplementary material for: Splice-Junction-Based Mapping of Alternative Isoforms in the Human Proteome
Source: Cell Rep. Author manuscript; Available in PMC 2020 Jan 15. (PMC6961840; doi:10.1016/j.celrep.2019.11.026)
Supplement: 2 [file NIHMS1546469-supplement-2.zip › DF1/PXD010154/Heart/Heart_28_TTN_KEEEVPPPPKEVTIMEEK.pdf]

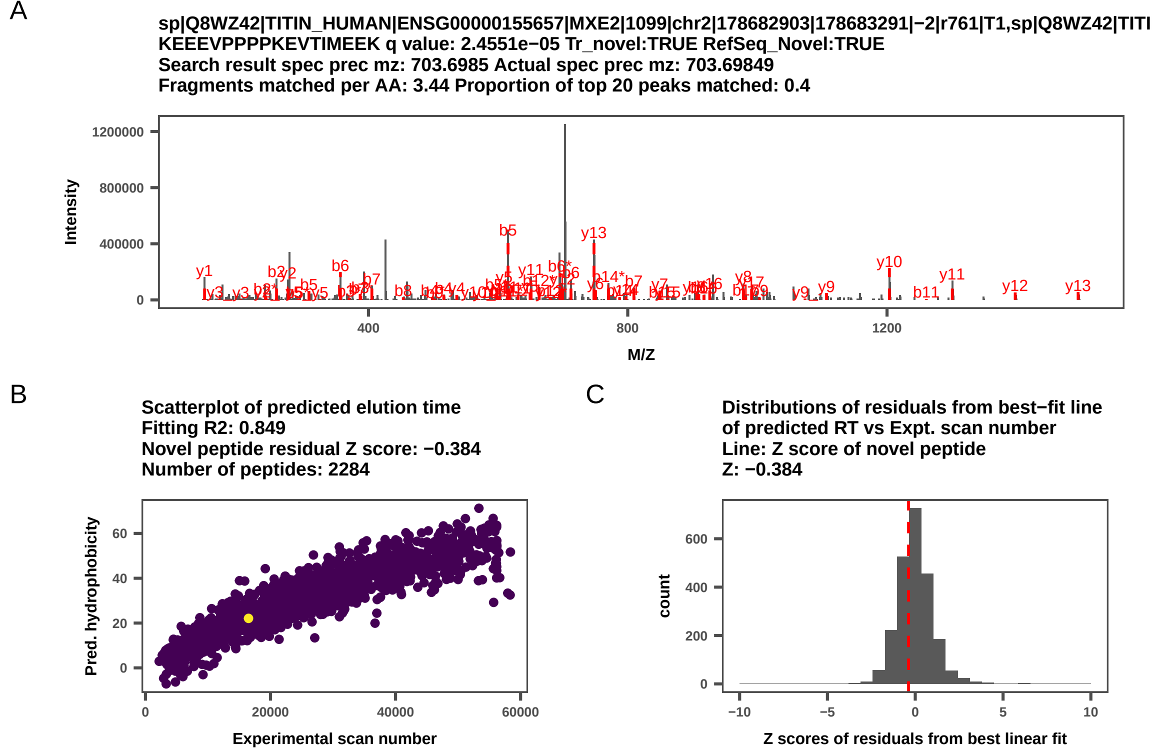
A
sp|Q8WZ42|TITIN_HUMAN|ENSG00000155657|MXE2|1099|chr2|178682903|178683291|−2|r761|T1,sp|Q8WZ42|TITI
KEEEVPPPPKEVTIMEEK q value: 2.4551e−05 Tr_novel:TRUE RefSeq_Novel:TRUE
Search result spec prec mz: 703.6985 Actual spec prec mz: 703.69849
Fragments matched per AA: 3.44 Proportion of top 20 peaks matched: 0.4
Intensity
1200000
800000
400000
0
400
800
1200
M/Z
y1
b6
b7
b5
y13
y10
y11
b11
y12
y13
y9
B
Scatterplot of predicted elution time
Fitting R2: 0.849
Novel peptide residual Z score: −0.384
Number of peptides: 2284
Pred. hydrophobicity
60
40
20
0
0
20000
40000
60000
Experimental scan number
C
Distributions of residuals from best−fit line
of predicted RT vs Expt. scan number
Line: Z score of novel peptide
Z: −0.384
count
600
400
200
0
−10
−5
0
5
10
Z scores of residuals from best linear fit
